# Improvement of Subthalamic Nucleus Deep Brain Stimulation in Sleeping Symptoms in Parkinson's Disease: A Meta-Analysis

**DOI:** 10.1155/2019/6280896

**Published:** 2019-10-08

**Authors:** Xue Zhang, Anmu Xie

**Affiliations:** ^1^Department of Neurology, Affiliated Hospital of Qingdao University, Qingdao, China; ^2^Neurological Regulation Institute of Qingdao University, Qingdao, China

## Abstract

**Introduction:**

The aim of this meta-analysis was to evaluate the effects of STN DBS on sleep quality and restless leg symptoms in individuals with PD.

**Methods:**

We searched the PubMed, Web of Science, EMBASE, CNKI, and WANFANG databases published between 1990 and 2019. The articles included were those that contained both pre- and postsurgery data acquired using International RLS Study Group criteria and the Pittsburgh sleep quality index (PSQI) questionnaire with patients' follow-up of at least three months. All studies that met the quality requirements were included in a meta-analysis performed using STATA 12.0 software.

**Results:**

Of 73 articles identified, 7 studies comprising 82 patients were qualified for the current meta-analysis. After adjusting for heterogeneity in study effect sizes, the random effects meta-analysis indicated that STN DBS improved sleep quality and restless leg symptoms significantly (SMD = −1.111, 95% CI: −1.918∼−0.304, *P*=0.007). Subgroup analysis showed that different sleep scoring criteria had different effects on the condition of sleeping after surgery.

**Conclusions:**

STN DBS is a powerful method in the management of sleep quality and restless leg symptoms in PD patients, but its long-term effects with larger populations must be thoroughly assessed.

## 1. Introduction

Parkinson's disease (PD), as a primary reason of morbidity and mortality worldwide, is a debilitating neurodegenerative condition characterized by motor and nonmotor symptoms among middle-aged and elderly individuals. As for treatment methods, currently, antiparkinsonian medication and surgical interventions have become mainstream [1, 2]. Generally, antiparkinsonian drugs, which are currently the first-line therapy for PD, are mostly used in patients during the early stage of the disease [3]. On the other hand, surgery using deep brain stimulation (DBS) is done during mid-stage to late-stage of the disease in patients with decreased sensitivity to available medicines or in patients with drug-induced complications [4].

Deep brain stimulation, which involves the application of electrical stimuli in specific nuclei, was clinically used in the late 1990s for the treatment of PD, and this technique has been widely and increasingly accepted during last 20 years. Currently, STN DBS significantly improves patients' primary motor symptoms as well as the quality of life, reducing their need for medications [5, 6]. The influence of STN DBS on nonmotor symptoms (NMS) of PD has received only a smaller amount of attention, even though recent records indicate that NMS occur in up to 100% of PD patients [7]. Nonmotor symptoms which include sleep disorders, cognitive deficiency, and autonomic deficiency are common and they all have a noteworthy effect on disability and subsequently quality of life [8]. Hence, it is imperative to frequently evaluate the development or deterioration of nonmotor symptoms in DBS patients.

Sleep disturbances are an integral aspect of the NMS of PD, which are common in PD patients, becoming increasingly more frequent and severe during the advanced stage of the disease [9, 10]. Lees et al. [11] conducted a national survey and found that more than 95% of advanced PD patients suffer from sleep disorders. Restless leg syndrome (RLS) is a sleep-related movement disorder characterized by the urge to move one's legs frequently accompanied by uncomfortable and unpleasant sensations during the period of rest or inactivity that interferes with the sleep of sufferers [12]. RLS can affect up to 24% of the adult population and may be increased in patients with Parkinson's disease (PD) [13]. Its pathophysiology is not known; however, it is supposed to be associated with diminished central dopaminergic transmission [14]. Reports are limited on the effect of PD surgery on RLS symptoms, and existing publications demonstrate conflicting results [15–18]. In this meta-analysis, we synthesized the results of published research on patient outcomes following bilateral DBS of the STN on sleep quality and restless leg symptoms and would be of great clinical value when considering patients and DBS target selection.

## 2. Materials and Methods

### 2.1. Literature Search

We selected the studies which investigated sleep quality and restless leg symptoms of patients with PD before and after bilateral DBS. An initial literature search was done by means of PubMed, Web of Science, EMBASE, CNKI, and WANFANG databases for the years 1990 to 2019. The search terms were (“bilateral deep brain stimulation” OR “bilateral stimulation”) OR (“bilateral stimulation” AND “subthalamic nucleus”) AND (“Parkinson disease” OR “Parkinson's disease”) AND (“sleep” OR “restless legs syndrome”). In addition, we searched the references of the recognized studies to find other satisfactory studies. This task was completed by two reviewers independently. When disagreements arose, a third reviewer was consulted.

### 2.2. Inclusion and Exclusion Criteria

The inclusion criteria were the following: (1) patients with idiopathic PD were treated with bilateral STN DBS, (2) the patients were followed up for at least 3 months, (3) presurgery and postsurgery data were obtained through International Restless Legs Syndrome Study Group (IRLSSG) criteria or PSQI questionnaire, respectively (RLS diagnosis was made according to established criteria including 4 essential features of RLS [19]. PSQI contains seven components with scores ranging from 0 to 21, and higher scores indicate worse sleep quality [20]), and (4) data were analyzed in the form of mean and standard deviation. The exclusion criteria were as follows: (1) preclinical studies reviews, meta-analysis, book chapters, letters to the editor, or case reports with no innovative data, (2) duplicated reports with identical data, (3) data from nonhuman species, and (4) insufficient original data.

### 2.3. Quality Assessment

Two reviewers evaluated the quality of the studies by means of the Methodological Index for Nonrandomized Studies (MINORS), which involves eight items. Each item was scored from 0 to 2: 0 representing that it was not reported in the article, 1 representing that it was reported but inadequately, and 2 representing that it was reported adequately [21].

### 2.4. Extraction

Two investigators reviewed the publications independently and extracted the applicable data from each qualified study, whereas objections were resolved by consulting a third reviewer. The below mentioned details were extracted: name of the first author, year of publication, sample size, duration of disease, time of following up, DBS programming, and the relevant pre- and posttreatment IRLS score and PSQI score concerning the STN DBS.

### 2.5. Statistical Analysis

STATA statistics software (version 12.0, USA) was used to analyze accessible data. Since two scales were used in our study, standardized mean difference (SMD), that is, Cohen's d, was used to estimate the size of the combined effect, with a confidence interval of 95%. SMD, as a standard statistic, was used to evaluate the comparisons of presurgery and postsurgery change. This value reflects an intervention-induced change of the outcome on an average and is used as a summary statistic in meta-analysis if the studies were measured in different ways. As for the heterogeneity of all the studies, Q-test and *I*^2^-statistics were used to evaluate the degree of it. An *I*^2^ > 50% or *P* < 0.05 designated significant heterogeneity, then a random-effects model would be used for meta-analysis [22]. Sensitivity analysis was performed by excluding each study and reanalyzing the remaining studies. Begg's test that measures funnel plot asymmetry was used to evaluate publication biases. A value of <0.05 for Begg's test was considered statistically significant [23].

## 3. Results

### 3.1. Characteristics of Eligible Studies

**Figure 1:**
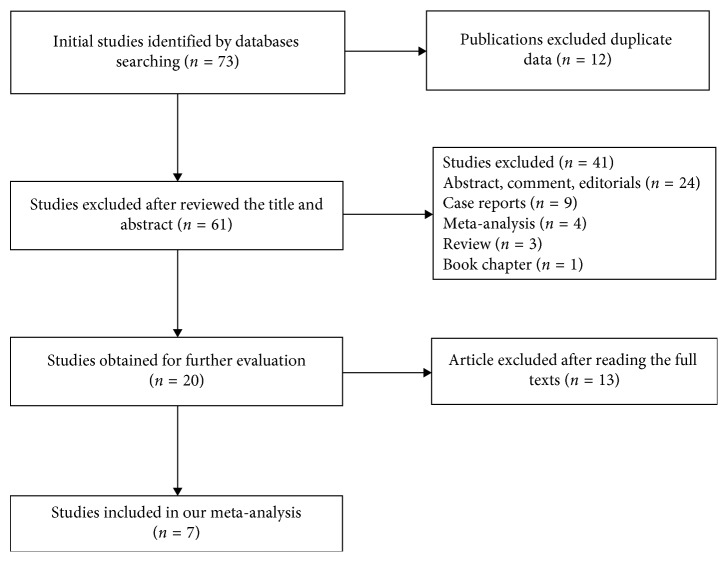
Flowchart used to include studies in the present meta-analysis.

We recognized 73 published studies, from which we omitted 12 because of the duplication. We further excluded 41 studies after evaluating titles and abstracts as they did not meet the inclusion criteria. After reading the full text of the remaining 20 articles, we omitted 13 studies due to lack of control groups. Finally, we had 7 eligible studies which fulfilled the inclusion criteria.  Figure 1 displays the flowchart of the screening method. All the included studies were follow-up type studies, with following up time ranging from 3 months to 12 months. The sample size was 82, and 45 patients were evaluated by means of the International RLS Study Group criteria, whereas 37 patients were evaluated by means of PSQI questionnaire. The PD patients involved underwent bilateral DBS before and after surgery. The main areas studied are described in Table 1. Quality assessment results from 7 included articles evaluated using MINORS analyses are presented in Table 2.

### 3.2. Quantitative Synthesis

**Figure 2:**
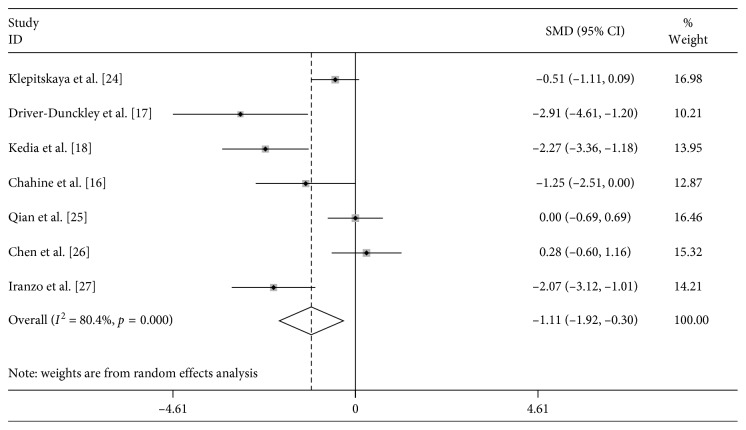
Forest plot for the change in sleeping symptoms observed presurgery and postsurgery.

Meta-analysis results of changes of RLS and sleeping after operation are displayed in Figure 2. The heterogeneity between the included studies showed that *I*^2^ = 80.4%; therefore, the random effects model was used to count the combined SMD. Based on the comparison of preoperative and postoperative change, we found that there was a significant change in the score among the presurgery stage and the postsurgery stage (SMD = −1.111, 95% CI: −1.918∼−0.304, *P*=0.007) (Figure 2).

**Figure 3:**
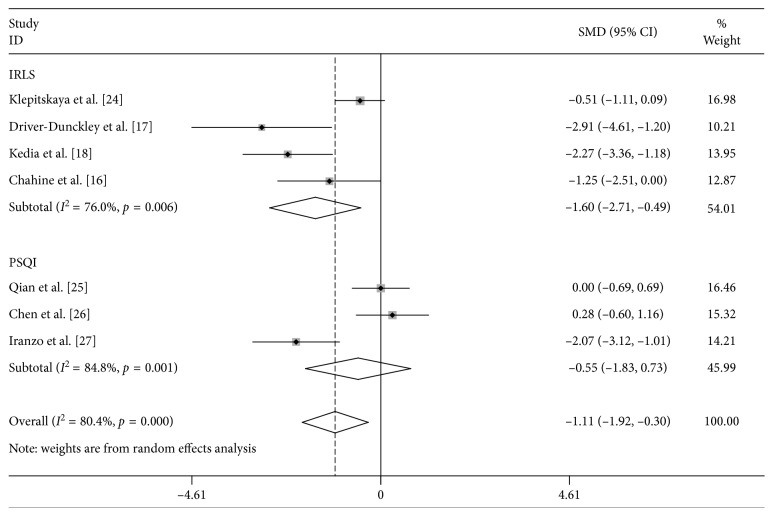
Subgroup analysis of the efficacy of STN DBS in the treatment of patients with RLS and sleeping symptoms.

Subgroup analysis was then conducted according to different scoring criteria. The results showed that the sleep quality of PD patients could be improved after STN DBS using IRLS scoring criteria (SMD = −1.598, 95% CI: −2.710∼−0.485, *P*=0.005), whereas no significant difference was observed in the PSQI score between the presurgery stage and the postsurgery stage (SMD = −0.551, 95% CI: −1.835∼0.733, *P*=0.400) (Figure 3).

### 3.3. Sensitivity Analysis and Publication Bias

**Figure 4:**
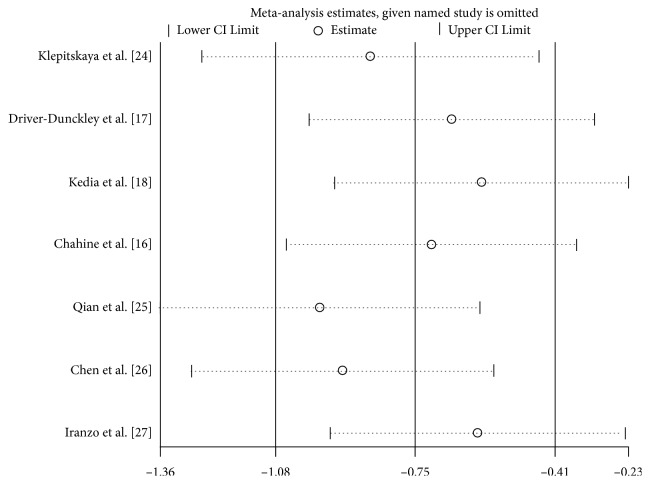
Sensitivity analysis of the summary OR coefficients on sleeping symptoms observed presurgery and postsurgery.

**Figure 5:**
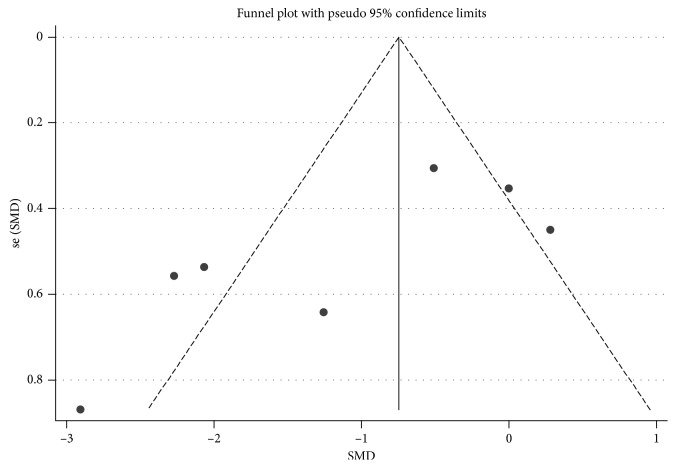
Funnel plot for publication bias in selection of studies.

In the sensitivity analysis, each study was omitted by turns to show the influence of every article contributing to this meta-analysis. No significant alterations were found in the pooled SMD, which showed a high level of stability of this meta-analysis (Figure 4). Begg's test was used to evaluate publication bias, and the funnel plot was approximately symmetric, indicating that there was no publication bias (*P*=0.293) (Figure 5).

## 4. Discussion

Deep brain stimulation (DBS) has become an attractive alternative for the treatment of neurodegenerative diseases. STN DBS has been well established over the past 20 years for the symptomatic treatment of motor symptoms and complications in advanced PD. It provides more constant and predictable benefits than pharmacological therapy. Numerous studies have documented that the majority of patients who undergo STN DBS achieve amelioration of motor symptoms and quality of life. Nonmotor symptoms (NMS) are nowadays recognized as an integral part of the clinical characteristics of PD, both at the early stages and throughout the whole course of the disease, and even at the very beginning of the disease, before any of the classical motor symptoms develop [28]. Many published meta-analyses have showed evidence for an adverse effect on cognition, depression, and apathy [29–31]. The present article is, to our knowledge, the first meta-analysis focusing on the effects of DBS on sleep quality and restless leg symptoms.

In particular, sleep disturbances in addition to neuropsychiatric symptoms (depression, cognitive dysfunctions, and psychosis) affect numerous PD patients. Sleep disruption is one of the most common nonmotor symptoms of PD and problematic in PD with estimates that as many as 90% of PD patients suffer from sleep problem [32]. There is substantial dissimilarity in the results of preceding studies using PSQI questionnaire on post-DBS sleep changes [25–27]. Moreover, previous studies have reported contradictory outcomes regarding the effects of STN DBS on RLS in PD. Kedia et al. [18] indicated that 11 PD patients out of 195 (5.6%) had the occurrence of problematic RLS after DBS STN, which is similar with Marques research that six patients out of 31 (19%) described postoperative onset of restless leg syndrome [33]. However, numerous studies have described improvement in RLS symptoms after STN DBS in patients with PD [18, 24, 34]. Klepitskaya et al. [24] established a significant improvement of the IRLS score and severity in 22 patients with PD and moderate-to-severe RLS after STN DBS. These improvements lasted for up to 2 years after surgery despite a significant reduction in dopaminergic therapy (34.5%–69.2%).

The present meta-analysis included 7 studies containing 82 PD patients comparing the differences in sleeping changes between presurgery and postsurgery patients. Through strict methodological and statistical analysis, our data suggested that there was a statistical significant difference in the scores between the presurgery stage and the postsurgery stage (SMD = −1.111, 95% CI: −1.981∼−0.304, *P*=0.007), which means that bilateral STN DBS did seem to improve the PD patients' sleeping condition. However, the subgroup analysis showed that different scoring criteria for sleeping had an impact on the condition of sleeping quality after surgery.

It is well recognized that several factors lead to sleep dysfunction in PD. Significantly, it has been established that degeneration of dopaminergic and nondopaminergic neurons underlying PD might have a part in PD-related sleep dysfunction, and if night-time awakenings at the end of a sleep cycle were followed by dystonia, prolonged periods of wakefulness will occur [35]. The sleep improvement induced by STN DBS has been explained by alleviation of nocturnal motor symptoms and reduction in doses of antiparkinsonian medications. In addition, it is possible that stimulation of the STN has effects on sleep regulatory centers by increasing slow-wave sleep as well as REM sleep but not altering the relative proportion of each sleep stage [36]. Enhancing the elimination of the possibly neurotoxic waste products that mount up in the awake central nervous system might also advance sleep functions [37].

In idiopathic RLS, low-dose dopaminergic therapy is effective, and chronic dopaminergic treatment can induce exacerbation and extension of RLS symptoms, called “augmentation.” This study showed that successful STN DBS was accompanied by a decrease of dopaminergic medication, which suggests that reduction in doses of antiparkinsonian medications may be an explanation for the improvement of RLS. Dopamine neurons are involved in the processes such as voluntary movement (Nigrostriatal system), cognitive/emotive functions (mesocorticolimbic system), and prolactin secretion (tuberoinfundibular system) [38, 39]. Dopaminergic pathology has been suggested to be related to the pathophysiological mechanism of RLS. Postoperative improvement of RLS is thought to be related to variations in basal ganglion neuron firing with downstream effects on the thalamus, which decreases the downstream disinhibition at the spinal level, thus alleviating abnormal sensations and motor restlessness [40]. Recent literature on the pathophysiology of RLS has implicated central dopaminergic dysfunction in several regions including the substantia nigra, striatum, putamen, and downstream disinhibition of the sensory dorsal horn and the intermediolateral nucleus of the spinal cord. DBS is a powerful treatment of dopamine-responsive symptoms of PD; therefore, it is reasonable to expect that DBS treatment can indirectly alleviate symptoms of other dopamine-responsive conditions, such as RLS.

This study provides evidence that STN DBS significantly improves sleep quality and restless leg symptoms at more than 3 months postoperatively compared to the preoperative baseline. There are several limitations of this article. First, the sample size of STN DBS was small, so additional studies are required to completely clarify the sleeping quality related to STN-DBS. Second, only the short-term effectiveness of DBS in treating PD was evaluated, thus, if our conclusion was suitable for long-term treatment was not clear. Significant heterogeneity was observed in this analysis. A potential explanation may be that relevant variables (e.g., sleep scoring criteria, published year, duration of PD, age, ethnicity, follow-up, etc.) in each study were diﬀerent.

## 5. Conclusion

In conclusion, STN DBS is a powerful method in the management of sleep quality and restless leg symptoms in PD patients. Further improvements, through basic research and methodological innovations, should make it applicable to long-term treatment of the disease. Considering the limitations stated above, further larger samples and multicenter cooperative studies in different populations should be carried out, and the precise intervention protocols for PD patients with different disease stages should be taken in future findings.

## Figures and Tables

**Table 1:** Characteristics of the eligible studies.

Author	Year	*N*	Age	Disease duration	DBS programming	Scale	Follow-up	Preoperative score	Preoperative LE	Postoperative score	Postoperative LE
Klepitskaya et al. [24]	2018	22	58.3 ± 7.4	10.2 ± 4.9	STN-DBS	IRLSSG criteria	6 months	19.59 ± 6.95	1203.2 ± 657.5	14.53 ± 12.27	370.8 ± 69.2
Kedia et al. [18]	2004	11	61	14.2	STN-DBS	IRLSSG criteria	6 months	15 ± 5.9	1169.4 ± 702.7	4.3 ± 3.1	294.0 ± 384.3
Chahine et al. [16]	2011	6	62 ± 8.8	13.1 ± 8	STN-DBS	IRLSSG criteria	6 months	23.0 ± 5.8	1193.8 ± 600.7	13.8 ± 8.6	599.6 ± 369.7
Driver-Dunckley et al. [17]	2006	6	65.1 ± 10.3	13.7 ± 3.1	STN-DBS	IRLSSG criteria	3 months	24.8 ± 8.3	1591.5 ± 536.3	4 ± 5.8	699.2 ± 509.4
Qian et al. [25]	2013	16	59.5 ± 8.5	8.9 ± 2.9	STN-DBS	PSQI	24 months	12 ± 3	756.5 ± 255.2	12 ± 4	420.8 ± 300.1
Chen et al. [26]	2011	10	59 ± 10	9 ± 3	STN-DBS	PSQI	12 months	11 ± 3	711 ± 253	12 ± 4	361 ± 206
Iranzo et al. [27]	2002	11	63.6 ± 7.8	17.3 ± 9.1	STN-DBS	PSQI	6 months	14.8 ± 4.5	—	5.4 ± 4.6	—

IRLSSG criteria: International restless leg syndrome study group (IRLSSG) criteria; PSQI: the Pittsburgh sleep quality index (PSQI) questionnaire; and LE: levodopa equivalents.

**Table 2:** MINORS scores of eligible studies.

Study	A	B	C	D	E	F	G	H	Total
Klepitskaya et al. [24]	2	0	2	2	0	2	2	0	10
Kedia et al. [18]	2	0	2	2	0	2	2	1	11
Chahine et al. [16]	2	0	2	2	0	2	2	1	11
Driver-Dunckley et al. [17]	2	2	2	2	0	2	2	0	12
Qian et al. [25]	2	0	2	2	0	2	2	0	10
Chen et al. [26]	2	0	2	2	0	2	2	0	10
Iranzo et al. [27]	2	2	2	2	0	2	2	0	12

A: a clearly stated aim; B: inclusion of consecutive patients; C: prospective collection of data; D: endpoints appropriate to the aim of the study; E: unbiased assessment of the study endpoint; F: follow-up period appropriate to the aim of the study; G: loss to follow-up less than 5%; H: prospective calculation of the sample size.
